# The messy coloniality of gender and development in Indigenous Wixárika communities

**DOI:** 10.1080/13552074.2023.2264638

**Published:** 2023-12-12

**Authors:** Paulina Ultreras Villagrana, Jennie Gamlin, María Teresa Fernández Aceves

**Keywords:** Coloniality, gender, development, Wixárika communities, Colonialidad, género, desarrollo, comunidades Wixárika

## Abstract

Understanding the coloniality of gendered lives, family dynamics, social arrangements, and political structures in Indigenous communities begins with confronting and interrogating a history written largely by and for men in positions of power. The archives are limited in terms of what can be gleaned about gender equality and what existed before the proliferation of European patriarchy. Indigenous Wixárika people tread a delicate balance between a lifeworld that is organised around a ritual–agricultural cycle, and the accelerating incorporation of the imperial mode of living and the coloniality of being, into their communities and culture. The ‘coloniality of gender’ explains how Indigenous women and men have been drawn into and shaped through contact zones, these sites of imperial intervention that have brought social, cultural, and structural changes to gender. Problematically, this concept assumes a one-way process of domination, whereby modern European power structures were imposed on Indigenous people. A critical exploration reveals how gender dynamics and equality were influenced by a much messier process, entangled with Wixárika’s cultural and religious systems as well as the leveraging of political collateral. This paper will draw on findings from a historical and ethnographic study of the coloniality of gender in Indigenous Wixárika communities. We will critically examine archival evidence alongside oral histories to suggest how social, development, and political interventions from the late 20th century challenge the idea of the ‘coloniality of gender’, and discuss how past and present actants collide and dialogue to bring about social change and greater gender equality.

## Abstract (fr)

Pour comprendre la colonialité des vies, des dynamiques familiales, des arrangements sociaux et des structures politiques axés sur le genre au sein des communautés autochtones, il faut d'abord confronter et interroger une histoire écrite en grande partie par et pour des hommes en position de pouvoir. Les archives sont limitées quant à ce que l'on peut apprendre sur l'égalité des genres et ce qui existait avant la prolifération du patriarcat européen. Le peuple autochtone des Wixárikas repose sur un équilibre délicat entre un « monde vécu » organisé autour d'un cycle rituel-agricole, et l'incorporation accélérée du mode de vie impérial (Brand et Wissen, 2021) et de la colonialité de l'être dans ses communautés et sa culture. La « colonialité du genre » explique comment les femmes et les hommes autochtones ont été attirés dans des zones de contact - ces sites d'intervention impériale qui ont entraîné des changements sociaux, culturels et structurels en matière de genre - et façonnés par elles. Le problème est que ce concept suppose un processus de domination à sens unique, par lequel les structures de pouvoir de l'Europe moderne ont été imposées aux populations autochtones. Une exploration critique révèle comment la dynamique et l'égalité des genres ont été influencées par un processus beaucoup plus désordonné, enchevêtré avec les systèmes culturels et religieux wixárikas, ainsi qu'à l'exploitation des garanties politiques. Cet article s'appuie sur les résultats d'une étude historique et ethnographique de la colonialité du genre dans les communautés autochtones wixárikas. Nous jetterons un regard critique sur les archives et les récits oraux afin de montrer comment les interventions sociales, politiques et de développement de la fin du XXe siècle remettent en question l'idée de la « colonialité du genre », et aborderons la manière dont les acteurs passés et présents se heurtent et dialoguent pour donner lieu à des changements sociaux et à une égalité accrue entre les genres.

## Abstract (es)

Entender la colonialidad de género, las dinámicas familiares, arreglos sociales y estructuras políticas en comunidades indígenas comienza con confrontar e interrogar la historia largamente escrita por y para hombres en posiciones de poder. Los archivos están limitados en términos de lo que puede vislumbrarse sobre igualdad de género antes de la proliferación del patriarcado europeo. Los indígenas Wixárika tejen un delicado balance entre la vida organizada en torno a los ciclos de rituales agrícolas, la acelerada incorporación de un modo imperial de vida y la colonialidad de ser en sus comunidades. La ‘colonialidad de género’ explica cómo mujeres y hombres indígenas han sido arrastrados y moldeados a través de zonas de contacto, sitios de intervención imperial que conllevan cambios estructurales, culturales y de género. Problemáticamente, este concepto asume una vía en el proceso de dominación, donde las estructuras de poder europeas fueron impuestas en los pueblos indígenas. Una exploración crítica revela cómo las dinámicas de género y la igualdad fueron influenciadas por un proceso mucho más turbulento, vinculado con los sistemas culturales y religiosos de los Wixárika y por una influencia política colateral. Mostramos hallazgos desde un estudio histórico y etnográfico de la colonialidad de género en comunidades indígenas Wixárika. Examinamos críticamente evidencia de archivo e historia oral para mostrar cómo las intervenciones sociales y de desarrollo, desde finales del siglo XX, desafiaron la idea de la ‘colonialidad de género’ y discutimos cómo personas del pasado y el presente dialogan para provocar un cambio social y mayor igualdad de género.

## Introduction

Understanding the coloniality of gendered lives, family dynamics, social arrangements, and political structures in Indigenous Wixárika communities in Jalisco State, Mexico begins with confronting and interrogating a history written largely by and for men in positions of power. The archives are limited in terms of what can be gleaned about gender equality and what existed before the proliferation of European patriarchy.

Joan Scott (1988) argued that the incorporation of gender as a category of analysis should elucidate the integral role that women have played in the historical process, rather than forming the basis of a specific chapter about women. More importantly, the analysis of gender helps challenge the invisibility, marginality, and subordination of women that went hand in hand with their exclusion from historical documentation. From this perspective, Scott not only addresses the absence of women in the past but also describes how history has been used as an arena for the creation of gendered knowledge. Recently, the historian Gabriela Aceves Sepúlveda, in her book *Women Made Visible*, convincingly argued that ‘In Mexico, as in the rest of Latin America, keeping files, creating archives, and writing history have all been conceptualised as masculine territory’ (Aceves Sepúlveda 2019, 7–8).

Archives reproduce the coloniality of gender by being empires and ontologies of imperial law (Premo and Yannakakis 2019). The colonial, state, and ecclesiastical archives that we have reviewed about Indigenous Wixárika (also known as Huicholes, pl. Wixaritari) communities in western Mexico reveal a colonising view that invisibilised women. Here we present the visibility or invisibility of gender relations as they appear in the archives and complement these with recent ethnographic data that explored women’s and men’s gendered experiences of social change.

This paper will analyse findings from a historical and ethnographic study of the coloniality of gender in Indigenous Wixárika communities. Coloniality of gender refers to the manner in which colonised men and women were drawn into modern Western societies on unequal terms, and their gender was defined intersectionally alongside their racialisation and ethnic differentiation. We draw on the rich debate on the conceptualisation and making of this theory by Latin Americanist decolonial feminists (Lugones 2010; Segato 2015) to examine critically archival evidence alongside oral histories. The coloniality of gender is a theory that is lacking in empirical foundations precisely because of invisibility. Women were largely absent from history at a time that saw the makings of human categorisations which shaped the power dynamics between men, women, and people of diverse origins, establishing from these hierarchical and universalising ideas about how nation-states and societies should function in the newly modern world. But this absence does not imply passivity, and our findings suggest that this was a complex and non-linear process, muddying the concept and idea of the ‘coloniality of gender’. Indigenous men and women actively negotiated the extent to which social, development, political interventions, and moral values would be incorporated into their village lives.

## Coloniality of gender

The ‘coloniality of gender’ explains how Indigenous women’s and men’s experiences have been shaped through colonial encounters. These contact zones, ‘spaces of imperial intervention’ (Pratt 2008, 8), brought social, cultural, and structural changes on unequal terms. This was a process that initiated with the colonisation of the Americas after 1492, a time in human history when invasion, trade, and migration brought together previously separated populations in large enough numbers. This significantly altered the global distribution of people and shape of human societies. It was the period in which modern Europe organised itself into nation-states and was redefined in relation to the world (Silverblatt 2009, ix).

The Atlantic shores became what Kelvin Santiago-Valles (2003, 48) refers to as the ‘nucleus of the (modern) world system’, the site of the first colonisation-globalisation and ‘initial site of the coloniality of power’. Aníbal Quijano (2010) describes how the colonial structures of power that emerged with the colonisation of the Americas produced specific forms of discrimination along racial, ethnic, and gendered lines. These were relational constructions that were assumed to be ‘objective’ and ‘scientific’ categories, that is, a ‘natural phenomenon’ (Quijano 2010, 22). The coloniality of power continues to ensure that the ‘dominated and discriminated against’ ‘are precisely the members of the “races”, “ethnies” or “nations” into which colonised populations were categorised, in the formative process of that world power, from the conquest of the Americas onward’ (Quijano 2010, 23).

It was around these structures of inequality that nation-states were built, and as governance became centralised, a new ‘public’ sphere emerged, along with its counterpart, the private space of ‘proper womanhood’ (Santiago-Valles 2003, 51), which became attached to connotations of inferiority, particularly in relation to domestic versus paid labour. This public/private division of space became central to the regulatory practices of the coloniality of power, implying the sanctioning of punitive surveillance and violence, and the containment and stigmatisation of practices that were carried out by men of power. This process is the coloniality of gender.

Maria Lugones builds on the ‘coloniality of power’ to conceptualise the idea and process of the coloniality of gender to make visible ‘what is imposed upon us’ (Lugones 2010, 742), with the intention of providing a way of ‘understanding, of reading, of perceiving our allegiance to this gender system so that we are in a position to reject [it]’. Gender arrangements need not be either patriarchal or heterosexual, yet this is the structure of the ‘modern/colonial gender system’ (Lugones 2010). Gender, under the coloniality of power, became one of the axes of oppression that permeated and controlled public and private spheres including sexual access, authority, labour, control of knowledge, and intersubjectivity (Lugones 2010, 744). This process was intersectional. Colonised women were racialised as inferior and violence, rape, slavery, and the ‘imposition of the heterosexual understanding of gender relations’ were all forms of domination. While Lugones’ framing is useful for understanding the different pathways of coloniality, like Quijano, her dichotomisation of coloniser and colonised suggests that this was a one-way process in which one system was imposed upon the other. This ‘damage-focused’ perspective (Tuck 2009, 412) frames Indigenous peoples as somehow depleted, defeated, or broken.

A damage or deficit perspective might suggest that Indigenous people had little agency, when in fact they actively engaged with authorities in a way that benefited their communities from the earliest stages of colonisation. Rita Segato (2015, 79) describes the coloniality of gender as they move from a communal ‘low-intensity patriarchy to the high-intensity modern colonial patriarchy’ that occurred as the *mundo aldea* (village world) was drawn into modern capitalist states. In this process, culture is ‘the cumulative decanting of history’, so that the customs and traditions of any group altered by its encounters with modernity become stabilised as their own ‘culture’, making each community or *pueblo* the historical vector or collective agent in a shared past and future (Segato 2015, 7). So that when modern ideas about gender begin to circulate in the *mundo aldea* they are drawn in selectively alongside existing cultural practices, melding with these.

As we will argue, it was not this black-and-white perspective, and in the case of Indigenous Wixaritari, colonialism was negotiated, ignored, and pushed back. From this perspective, we go further in this debate on the coloniality of gender by analysing it from historical evidence.

## Context and methods

The authors of this paper are anthropologists and historians whose career focus coincides with gendered historical accounts of communities in the north-west of Mexico. The authors’ expertise includes a longstanding ethnographic trajectory with Indigenous Wixárika communities using the methods of feminist ethnography, historical expertise on the relationships in northern Jalisco State between rural cattle ranchers and Indigenous Wixárika communities, and theoretical approaches to gender in history and the gendered political history of the early 20th century. The challenge was to write from the historical and anthropological perspective, combining and respecting the methods of each discipline, and giving relevance to a long-term analysis, precisely that seems to be the great contribution of this article. This combination has enabled a rich and continual dialogue between gender and history, archive data and ethnography, Wixárika and mestizo historical dynamics, theory, and daily life in our collective data analyses, which in many ways reflect the ongoing tensions experienced in Wixárika communities through history as different ways of life were negotiated, pushed back, and shaped contemporary realities.

Wixaritari inhabit a region of the Sierra Madre Occidental in north-western Mexico that until the 1980s was not accessible by vehicle and only in very recent years has been served with electricity. Government services including schools and health care were virtually non-existent until the Plan HUICOT (Huichol-Cora-Tepehuan; see discussion below) was initiated, so many of the older generations are monolingual Wixárika speakers. Wixaritari have always traded and migrated for work and as their highland home was on the salt route, they have never lived in isolation. Straddling the states of Jalisco, Nayarit, and Durango, their territory is politically divided into three semi-autonomous communities (Santa Catarina Cuexcomatitlán/Tuapurie, San Andrés Cohamiata/Tateikie, San Sebastián Teponahuaxtlán/Waut+u) as well as many individual villages. Since early colonialism, in the 16th century, their highland communities have been visited by missionaries, travellers, and later on anthropologists and government officials. We explored this past from a gender perspective through historical and ethnographic research. Among the archives we visited are the Historical Archive of Jalisco, the School Archive of the Department of Public Education in Jalisco State, the Franciscan Archive, the Historical Archive of the Secretary for Health, and the Archive of the National Indigenous Institute. We also reviewed the reports of missionaries, colonists, and envoys, as well as the more recent accounts by travellers and anthropologists including Carl Lumholtz (1902 [1895, 1898]) and Robert Zingg (1938 [1934]). Almost without exception, save for the short reports written by female teachers in the early 20th century located in the School Archive, these are records of encounters written by men, for men, based on conversations with men.

We complemented archive data with oral histories gathered between 2021 and 2023 using the methods of feminist ethnography (Davis and Craven 2016), implying that a range of ethnographic methods including observation, in-depth and semi-structured interviews, and field diaries were used to explore gendered power dynamics and elicit a gender-specific focus on historical experiences. In addition, we interviewed government employees, anthropologists, and health workers who had spent time in Wixárika communities over many years and during changing circumstances; these include conversations with Leopoldo Lopez^1^ (regional welfare programme co-ordinator). Both archive and ethnographic data collection were disrupted by COVID-19. While some archives would not permit in-person visits, others were closed until late in 2022. It was also impossible to visit the Wixárika communities, which were at one point entirely closed to external people in order to prevent the virus from entering. Once this restriction had been lifted it became apparent that the region of northern Jalisco that we needed to visit had become a hotbed for drug-related activity while Mexico was in lockdown, making transit into the communities potentially hazardous. Longstanding Wixárika collaborators who have been working with the project Principal Investigator (Gamlin) for many years themselves had to challenge their communities’ gender expectations in order to carry out the ethnography, resulting in incredibly rich interviews. All interviews with Wixaritari have been anonymised for confidentiality. In the pages that follow, we will give a critical analysis and interpretation of these data, constructed by continually asking: what does this tell us about gender and how have these historical events and processes impacted the coloniality of gender and development? We examine three aspects of this process: how negotiations between the state and communities happened man-to-man, the increasing visibility of girls and women through educational archives, and the developmental indigenous policies of the late 20th century that have brought significant changes in gender relations.

The Wixaritari did not exist as a separate ethnic group when Mexico was colonised, and we know little about the gender relations of their predecessors*.* Archaeological evidence from central Mexico suggests that women were held in high regard, their position in the community sacred because of their fertility. In his paper on ‘politicised maternities’, Jaime Delgado Rubio (2022) argued for the denaturalisation of the idea that in precolonial societies women were confined to the private sphere. Archaeological evidence suggests the contrary, that in fact the maternal role may have held political and ritual authority. Gender ideas shaped physical and social behaviours and spaces, as they do now, but it is most likely that the idea of a separation between public and private, with the former and its associations of greater importance than the latter, did not exist.

When the first missionaries began to arrive in the 16th century, Wixaritari responded to colonisation by negotiating, adopting, selecting, reinterpreting, and discarding modern society and its norms and morals. A critical exploration reveals how gender dynamics and equality were influenced by a messy process, entangled with Wixárika cultural and religious systems as well as the leveraging of political collateral through which a new gender order would be established.

Colonial domination was not imposed but brokered, and we identified three historical periods where relationships between state and communities were differentiated in terms of governance and power, broadly speaking these were (1) Spanish colonisation (15th to early 19th century) when Wixaritari were drawn into the conquest as militia, and missionaries attempted to establish the modern institution of Catholic heterosexual marriage in Indigenous communities; (2) 19th century independent and ‘liberal’ Mexico which saw the institutionalisation of private property and the private/public domains; and (3) post-revolutionary 20th century and modern state, in which negotiations and ties were modified, and when education, health, and welfare programmes were embedded in Wixárika lands.

## Negotiations man-to-man

**Figure 1.**
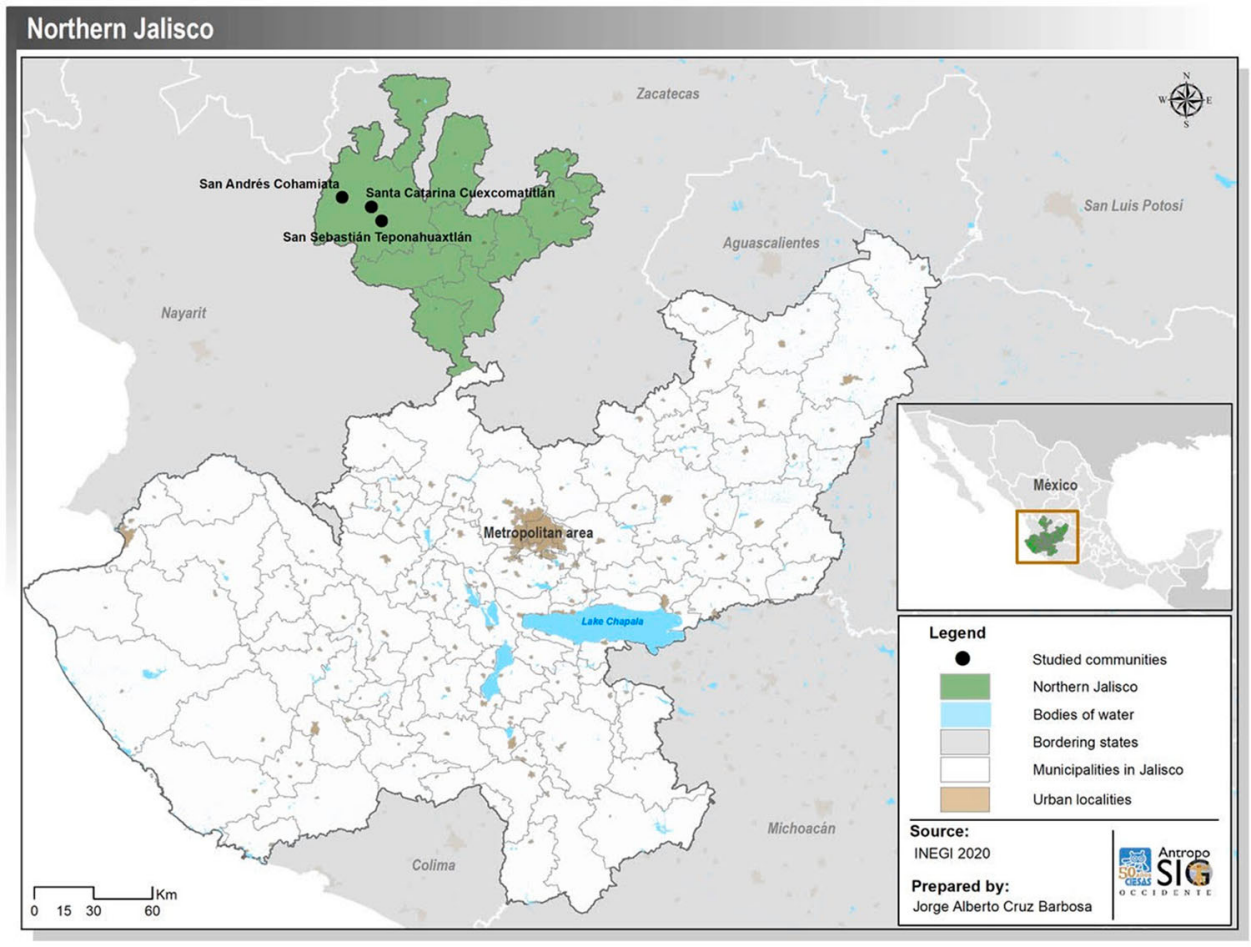
Map of Jalisco State showing northern Jalisco and wixaritari communities.

Since the beginning of colonisation, Spaniards attempted to impose the European gender system and erase the complementary gender roles (Kellogg 2005). The historical accounts tend to refer mainly to the negotiations man to man in relation to warfare and the granting of land. This evidence concurs with Santiago Valles’s argument that the governance became centralised from men to men, while women most of the time were invisible because, supposedly, they were in the private sphere. The colonisation of this portion of the territory was difficult for the Spanish because of intense resistance on the part of the local Indigenous population. In colonial chronicles, the inhabitants of this region were generically referred to as ‘*chichimecas*’, a term that grouped together ethnicities (Álvarez 2016) and considered by colonisers to be uncivilised barbarians, and savages (Arlegui 1851 [1737]; María and Guillermo 2003 [1575–1580]; Tello 1984). These characteristics became associated with the use of land in the region: dispersed settlements of semi-nomadic groups, which contrasted with the Western idea of the right of possession and ownership in towns or cities. The discovery of silver mines in the state of Zacatecas in the second half of the 16th century brought an urgency; the conquest and domination of this territory as an extraction route between the cities of Guadalajara and Zacatecas was needed. Violent encounters between colonisers and Indigenous groups led the Spanish Crown to opt for diplomacy and by the end of the century, in alliance with the Tlaxcaltecas (a central Mexican Indigenous group), the colonial government formed the *Gobierno de las Fronteras de Colotlán*, a jurisdiction inhabited by the various ethnic groups including the Wixaritari willing to cease attacks and protect the silver route from Indigenous rebellions. In exchange these groups, known as *Indios Flecheros*, were given the right to carry arms, ride horses, tax relief, and exemption from forced labour in the mines (Álvarez 2016; Güereca Durán 2019). Service to the Crown as a *flechero* Indian throughout the colonial period earned them protection for their lands and natural resources. By the 18th century, we argue, many *flechero* Indians no longer recalled how they had obtained these privileges and appealed for land rights based on having possessed them for ‘time immemorial’ or by ‘custom’ (community norms). These were rights or privileges that were fought man to man, gendering political relationships and establishing male authority (Figure 1).

Although the Spanish presence was infrequent and local autonomy continued, cross-cultural encounters after the conquest became the foundation of new colonial legal institutions that Indigenous communities used to their advantage. Yannakakis (2023, 7), in her research on Oaxaca State, argues that such litigations became ‘a dominant feature of colonial life, and a primary mode through which native peoples sought to protect old privileges and rights, or advocate for new ones’. These negotiations overwhelmingly occurred man to man: between male Indigenous leaders and male colonial administrators (Yannakakis 2023, 7). For Wixárika women, this became a double-edged knife, as while Wixárika men gained power to challenge the colonial state, aiding the retention of their customs and religious organisation, they also gained power over women both legally and customary.

In parallel to legal and political colonisations, from the 16th century onwards Franciscan missionaries visited Wixárika communities with the primary objective of instilling the idea of Catholic marriage in the minds and hearts of Wixaritari, but with limited success. While the use of whipping had been introduced as a form of punishment and was used abundantly, Wixaritari were reluctant to be converted to Catholicism, and both men and women resisted either abandoning their own religious ceremonies and rituals or adopting practices of christening, formal marriage, and strict heterosexuality. In 1853, the Friar Felipe de Jesús Muñoz notes how the missionary of San Andrés describes the church abandoned and ‘among their sins, sodomy predominates’. A few years later it was proposed that to evangelise the Huichols ‘ceremonies should not be forbidden, nor sacred sites destroyed but … their customs can be gradually pulled out of their minds until no trace remains’ (Muñoz 1853), suggesting that the imposition of a modern and Catholic moral and gendered order did not happen. However, little by little some moral ideas were incorporated into Wixárika customs while Catholicism melded with their own religious mythology, but this was tempered by a firm grip by women and men on their Wixárika way of life.

## Independent Mexico: the institutionalisation of patriarchy and private land ownership

After independence in 1821, the Mexican government made legislative changes aimed at gaining greater control over Indigenous populations so that a private land regime could be installed. These changes were not instantaneous but by the latter part of the 19th century, the Mexican ‘liberal’ state began promoting ‘freedom of property, not freedom of persons’, with the patriarchal family acquiring a greater political significance (Dore 2000, 9). The privatisation of land and secularisation of society altered gender relations (Law 1856 in Labastida 1893) and reinforced the subordination of women to patriarchal authorities. In the process of land privatisation, women ‘were excluded from the general expansion in personal rights’ (Dore 2000, 18). At a local level, however, the Wixaritari used their autonomy to retain communal land arrangements.

This complex set of relationships between Wixárika male authorities, the colonial state, and institutions such as the Court of Indians which had enabled litigation for Indigenous communities, also oversaw the gradual transformation of Wixárika social organisation into a patriarchal gender order. However, we hypothesise that this was a complex dynamic within which Wixaritari selectively adopted aspects of the modern and Catholic orders in such a way that they did not threaten their native set of customs and traditions. While their use of litigation based on customary law at the hands of men established male political authority within their communities, the privileges that they gained as *indios flecheros* for the Spanish Crown as well as their geographical location high in the mountains, possibly enabled them to conserve central aspects of their agricultural–religious structure in which women held equal or superior value to men.

In independent Mexico, local political autonomy meant Indians elected their own (male) community authorities, who were renewed annually through an internal selection process. Indigenous authorities could punish minor and civil offences such as marital infidelity or other crimes of a sexual nature (Zingg 1938, 134), but in cases of criminal offences the offender should be handed over to municipal authorities (Diguet 2005, 40–1). Relations outside the community, with the state, followed a patriarchal structure: negotiations were always between Indigenous men and the male state representatives. In Wixárika communities, authorities comprised governor, mayor, captain, and sheriff, each with *topil*, or assistant. Women known as *puyustes* or *tenatzi* were also chosen and made responsible for the care of objects of worship (Diguet 2005, 102), also occasionally occupying the role of *topiles.* Zingg (1938) and Lumholtz (1902) both discuss how *tenanzi* or *tenanchas* were originally appointed as caretakers or assistants to the senior officers and on occasions also their mistress, as was frequently the case in mestizo towns. In the three decades that passed between Lumholtz’s visit and the year that Zingg spent in the Sierra, the role of the *tenancha* had morphed into the institutionalisation of polygamy, where customary law allows for men to marry two or more women (Zingg 1938, 136). Polygamy is now less common but continues to be acceptable within the confines of customary law. This institutionalisation of bigamy may reflect the more open attitudes to sexuality that have prevailed despite the presence of Catholic missionaries. As noted by both Lumholtz and Zingg, both men and women had far more sexual freedom at their time of writing (1890s; Zingg 1938) and although lip service may have been granted to the Catholic institution of marriage, this did not reflect a moral conviction. As Segato (2015) remarks, communities and places become the cultural vectors for history. In this case, what started out as a liberty taken by officials or powerful men in Spanish-speaking towns became a Wixárika practice backed up by customary law and unchallenged by national legislation.

Although relations were man-to-man and the distribution of land privileged men, at a communal level women held some responsibilities and rights (Mallon 1994). Wixárika women continued to play a central role in ceremonial activities on an equal basis as men, as they had always done, and in contrast to the patriarchal orders of the Catholic Church, it was not unusual for there to be female shamans or religious leaders.

Indigenous scholar Jaimes* Guerrero (2003) uses the term ‘trickle-down patriarchy’ to describe the process through which patriarchal patterns of social organisation, in particular male dominance in politics, are adopted by Indigenous communities. Indigenous governance within the colonial and postcolonial state structure is patriarchal and these structures were required by the state as a means of exercising control over native populations. This happened on a par with racialisation as colonial powers took control away from native men and women, but gave it back to native men in the form of control over women. These forms of social organisation then permeated male–female gender relationships, creating a gender asymmetry that could well be the origins of high levels of violence against women (Gamlin 2020; Segato 2015). We know from Franciscan friars and works of both Zingg and Lumholtz that public corporal punishment, including whipping and the stocks, was used for civil crimes such as adultery, and we hypothesise that this sanctioning of physical violence by male authorities may have bled into interpersonal dynamics as in national legislation, women became the property of men. But these processes of patriarchal domination were mediated through and would have come up against the high status of women in religious and rituals practices. There is little evidence of how Wixárika women responded to political re-ordering in the 18th and 19th centuries, but this changed rapidly in the latter part of the 20th century when formal education and development infrastructure brought significant changes to their way of life.

## Post-revolutionary and modern Mexico: education opens door to girls and women

While negotiations over land took place man to man, in attempts to modernise the nation in the late 19th century, girls and women were included in educational projects, although following a patriarchal axis. The ‘place of proper womanhood’ was to be modernised and notions of hygiene, and care for the home and children, were incorporated into national teaching programmes (Vaughan 2000). These reinforced the idea of the public/private division of space and emphasised the gendered separation of tasks.

Education was delivered in the Sierra by diverse institutions from the 19th century onwards. The municipalities of Jalisco brought the first teachers in the late 1800s, followed in the 20th century, and finally, through the *Secretaría de Educación Pública* (SEP), a bilingual system of education in the latter part of the 20th century.

In 1887, an education project was proposed that would ‘promote as much as possible the Indigenous class, so that through enlightenment they rise the state of prostration and dejection in which they find themselves’ (Official Correspondence 1887). This project was part of 19th-century liberalism that sought to integrate Indigenous people into the national state to save the ‘inferior’ races, considered obstacles to social progress. Consequentially, different social and economic reforms were proposed to integrate them into the nation (Isais Contreras 2018). This new colonisation saw education as a synonym of advancement, where children were educated according to their gender, with boys taught by men and girls by women, entrenching the modern gender order.

In 1888, schools for boys and girls were established in the three main communities: Santa Catarina, San Sebastián, and San Andrés (Correa y Chacón 1888). Finding teachers was a complicated task as mestizos (persons of mixed European and Indigenous ancestry)^2^ found it a challenge to settle in Indigenous communities with the Wixaritari reluctant to permit their residence, possibly as a direct means of resisting the imposition of educational programmes. However, in April 1888 Francisco Minjares, from the Wixárika community of Santa Catarina ‘who has “some knowledge” that he had acquired in the Colegio de Zacatecas’ (Correa y Chacón 1887) was appointed.

A year later, a school reform was ordered by Pérez Verdía, making the case that most of the inhabitants of the municipality where ‘they do not speak Spanish, they walk almost naked, they feed only on roasted corn, they live in the countryside, they do not know the indissolubility of marriage and they profess idolatry’. In general, the reformers perceived that Indigenous people were little likely to ‘give up their habits’ (Pérez Verdía 1889). The dominant belief among influential officials, of the time including the eminent Jaliscian lawyer and policymaker Pérez Verdía, was that Indigenous peoples should be incorporated into the project of the Mexican nation and ‘civilised’ through education. However, the Wixaritari who attended Franciscan missionary and hospice schools in cities did not incorporate their new learnings upon returning to their communities. On the contrary, they returned to use their traditional dress and ‘old customs’. Faced with this situation, mestizo teachers were brought in to establish two central schools, one for boys and one for girls, both in the community of Santa Catarina. The boys’ school came under the direction of a ‘committed male teacher’ and an assistant Wixárika principal. With space for 24 boys, the school operated on a boarding basis and care was taken that children from all the communities attended. From the very beginning fewer girls than boys could be educated and the girls’ school, run by a female principal only, had capacity for 15 children (Pérez Verdía 1889). This precedent defined girls’ school attendance until the end of the 20th century.

From 1890 teaching was led by mestizos with no or little knowledge of Wixárika. Education was in Spanish and omitted any teachings on traditional knowledge or ways of life linked to the Indigenous past. The educational project followed the precepts established by Pérez Verdía, ‘good customs’ should be taught, certain moral values, work, and hygiene, to eliminate characteristics considered a hindrance of civilisation. Importantly, teachers became the intermediaries between the state and Indigenous communities.

In their reports to the board, teachers highlighted the difficulties they had in educating children. In 1902, the director of the girls’ school in Santa Catarina, Cruz Robles, also reported that educational work was difficult because parents did not require their daughters to go to school, hence girls were at home and did not learn to speak Spanish. Boys and girls in the first and second grades were instructed in arithmetic, national language (Spanish), morals, writing, gymnastics, and singing. In addition, boys received lessons in geometry and military exercise (Robles 1902).

In their reports, teachers insisted that another obstacle to progress in education was the constant absence of children from classes, despite the insistence of teachers on inviting parents to send their children to school. One of the reasons for the non-attendance of children was because they helped their parents in agricultural work. To this day, the agricultural calendar and civic calendars are different, as Fausto Bautista (pseudonym), an elder of the community of Santa Catarina whose grandfather had been one of the first Wixárika teachers, recalled how ‘the Wixaritari had their own calendar and the only calendar that has survived is that of the ceremonial centres, because they take their ceremonial activities based on rain’ (interview, Tuapurie, November 2021).

After the Mexican Revolution of 1910, the new revolutionary state reinforced a patriarchal structure in different realms of society, institutions, legislation, and policies. A significant change came in 1946 when schools ceased to be gender-segregated, in line with national education policy. Teachers used civic holidays to insert themselves into a community that was reluctant to accept them, education, and modernisation (Vaughan 1994). By this stage, many teachers were female and their interactions with mothers in the community around their children’s education and civic events (Mothers’ Day, Independence Day) began to change Wixárika women’s positioning once more.

Over the course of 50 years, teachers reported different concerns. While at the end of the 19th century they had emphasised the ‘barbarism’ of students, 50 years later the focus was on the advantages of education and its incidence in the reduction of crimes. In the 1940s, the number of children attending school increased, and teachers less frequently reported ‘barbarism’ or ‘backwardness’. This is more likely a reflection of how teachers’ understanding of Indigenous people changed through interactions with them than a significant shift in Wixárika cultural practices – although acceptance of teachers also grew as time went on.
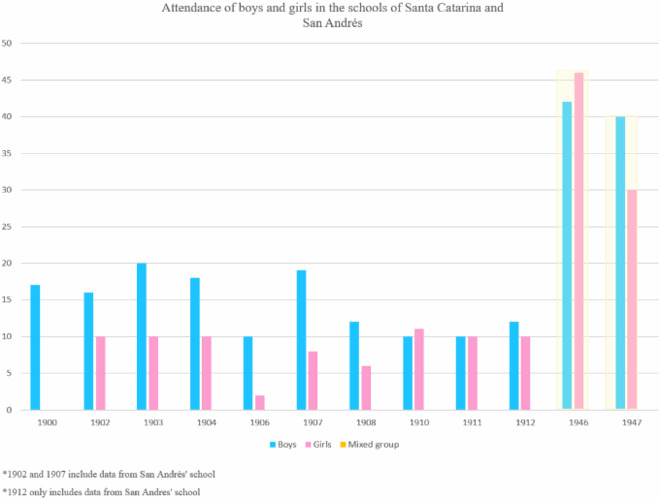


The following graph tracks the trend in the number of boys and girls who attended schools in different years between 1906 and 1947, highlighting that fewer girls went to schools than boys from 1900 to 1911, while from 1946 we observe a significant increase in the attendance of both boys and girls. Possibly due to disruptions caused by the Mexican Revolution (1910–1917) and the Cristero War (1926–1929), we found no information on education from 1913 to 1945. The increase in school attendance was part of gendered educational policies that were put in place seeking the modernisation of patriarchy. Women remained second-class citizens, subject to male domination, but with roles in the domestic and social spheres that would assist the state as mothers in the modernising process, of education, health, morality, and child protection. In the *ejidos* (communal lands), men, women, and young people should have access to schools and medical services. Although state-level policies such as these did not reach Wixárika communities, their establishment as hegemonic forms of social organisation had reverberations in all their interactions with state institutions (Vaughan 2000).

Nathaniel Morris (2017) analyses SEP’s ‘civilising’ programme that took place from 1920 to 1940 in the Wixárika highlands and highlights its failure as a programme that aimed to assimilate Indigenous people, through education, into the Mexican nation, linking this to discrepancies between official government policies and their local-level implementation. The ideologues of the SEP proposed a mestizo ethnocentric curriculum and the eradication of indigenous identity, language, and culture. For the Wixaritari, schools were the beginning of the end of their political autonomy, language, and ethnic identity. As Fausto Bautista explained, people would say ‘with what you are doing [setting up schools] you are going to sell us to the *teiwarixi* (mestizos)’ (interview, Tuapurie, November 2021). However, according to Morris (2017), the educational project of the SEP failed. Teachers left *en masse*, not returning until the launch of the Plan HUICOT (Huichol-Cora-Tepehuan), a federal development policy in the late 1960s.

Fausto Bautista recalled how ‘soldiers came to recruit children to study in a school called “The Children of the Army” that had been created in Guadalupe Zacatecas … in the time of Lázaro Cárdenas [1934–1940]’ (interview, Tuapurie, November 2021). On their return, these children were expected to become educators in their communities and Bautista commented on how, from the 1950s, Wixaritari teachers increasingly asked parents to send their children to school. While schools were mostly run and accepted by Wixaritari, with girls attending in higher numbers, the population remained divided, and Alicia Robles (pseudonym), a female elder from Santa Catarina, recalls her father saying ‘I had my children so that they can help me, not so that they can be taken by the *teiwarixi* (mestizos), one day they will sell our community’ (interview, Nueva Colonia, 17 August 2022). This post-war period, about which there is little documentation may have been pivotal in defining gender relations. Boys who were sent to school outside the highland would have returned with the mestizo idea of what it is to be a man in Mexico instilled in them. This was a time when gendered roles were firmly established along the lines of a breadwinner model of patriarchy. Legally, women were still the property of men and household as the ‘proper place for women’ was reflected in modern institutions. Ideas of gender equality, women’s empowerment, and investment in maternal and reproductive health services for women had not yet emerged.

In the latter part of the 20th century, Mexico’s development required education, infrastructure, and economic growth and it was with these aims that the Plan HUICOT was developed, comprising the building of five boarding schools, four day schools, and five health centres (Programas en acción 1972, 18). Vehicle access was also cut into the mountainside and airstrips were habilitated in each of the major towns. This was the beginning of major social and cultural changes as roads made it easier for men and women to travel in and out of their communities and Conditional Cash Transfer programmes such as *Prospera* (2014–2019) and *Oportunidades* (2002–2014) pushed cash into women’s pockets. Small businesses began to open, and the social and geographic landscape changed at a faster pace than ever before.

Leopoldo López, who was director of the National Indigenous Institute in Mezquitic from 1998 to 2009, shared with us how the Plan HUICOT programmes were intended to ‘develop’ the four Indigenous regions that they spanned. It was many years though before the programme leaders realised that so long as the Wixaritari did not feel ownership of these programmes, they would be unsustainable. Wixaritari were mostly excluded from training or knowledge transfer until the close of the millennium and López remarked on the continuity of a pattern that had been established during the colonial period: relationships between the state and Indigenous community were still man-to-man (interview, Usmajac, 25 October 2022). It was not until one woman from San Andrés Cohamiata, who went by the name of Trinidad Reza, spoke up that women were finally invited to co-ordinate the Community Planning Committee in the early 2000s. Santa Catarina and San Sebastián soon followed suit and began to incorporate women into the decision-making process. One of the most significant factors to influence womens’ participation in the politics and running of their communities was the arrival in the year 2000 of a campus of the University of Guadalajara, in the municipality of Colotlán, in northern Jalisco, facilitating women’s access to higher education as their tendency to begin motherhood at a young age made studying beyond their communities almost prohibitive. Education brought about change for both girls and boys. Alicia Robles explained that it was thanks to education that women are now better able to defend themselves, they are no longer beaten by their husbands, but, she argues, the change came from the mestizos, ‘the phrase “the woman has the same values as a man” is from the *teiwarixi* and they have trained women to defend themselves’ (interview, Nueva Colonia, 17 August 2022).

In the field of health, nurses and doctors became intermediaries between the community and the state, and the clinic became something of a government outpost. From the 1970s, the presence of the Ministry of Health, through the health centres, was crucial. In the early years they took advantage of the concentration of Indigenous people in their customary festivities (*cambio de varas*/change of office ceremony, Semana Santa/easter week, fiesta del maíz/maize harvest and religious festivity) to carry out consultations, talks, and health promotion. The objectives of bringing health services were to educate the population on ‘customs that were beneficial to health’, offer medical services, and to programme community development activities (Acción indigenista 21–2). Leopoldo López indicated that there was greater acceptance of health services thanks to the work and strategic employment of Wixárika female health promoters. Their functions included translation and support for doctors (washing, cleaning, cooking – all activities that have been linked to the ‘space of proper womanhood’). These women became intermediaries between the Ministry of Health and the community. Later, many Wixárika women trained as nurses, also playing an important role in community acceptance of medical services, in giving confidence to the sick and using health services, as well as raising people’s awareness, for example, on the importance of vaccinating children and having their cervical screening test. This opened the door for women to begin holding political offices in their communities. In agreement with Leopoldo López, Fausto Bautista commented that it was representatives of the Mexican state who encouraged the incorporation of women in community decision-making, ‘in those meetings it was said that with a woman commissioner there could be an improvement in terms of corruption’ (interview, Tuapurie, November 2021). In December 2022, the first ever female commissioner was appointed in the community of Santa Catarina. It is yet to be seen how this will impact on gender or on corruption.

## Conclusions

Indigenous Wixárika people tread a delicate balance between a lifeworld that is organised around a ritual–agricultural cycle, and the accelerating incorporation of the ‘Imperial mode of living’ (Brand and Wissen 2021) into their communities and culture. Since the arrival of the Spanish in the 16th century, Wixaritari have been resisting and negotiating with colonial power. Archival research makes visible the contacts between male Indigenous Wixárika with male colonial representatives of the state and Church, and we can trace through history how some of these negotiations happened and what they have meant for communities. The absence of women from these same archives makes it difficult to assess what role they played in this same process, but gender is a category of analysis that encompasses men and women, and a critical analysis of visibility and invisibility gives some indications of how the messy process of the coloniality of gender played out. The privileges that Wixaritari gained early on in the colonial process have had a lasting impact on their culture. To this day, Wixaritari are known for being one of the Mexican communities who have most fiercely guarded their customs and religion. It is possible that in this process they firstly protected and then later undermined the role of women.

Following Segato’s framing of the *aldea* or village as a historical vector into which culture is decanted, Wixárika political-autonomy enabled them to retain the ordering of their communities around an agricultural–religious structure that is reproduced in and reproduces mytho-history of their communities. Evident in this structure is the central role of women who are venerated for their reproductive capabilities and share a parallel role in religious activities. However, as patriarchy becomes embedded, at an ever-increasing rate, in their communities this too becomes part of their own history. Men as community leaders were given the authority to police this and to institutionalise their dominance in ways that reflect some of the highly gendered unequal values that they have learned from both the Mexican state and Catholic moral teachings of missionaries, disadvantaging women in both the personal and political arenas. The coloniality of gender is evident too in the trickle-down patriarchy that saw how Wixaritari male office holders replicate the patriarchal forms that are bestowed upon their relationships within the community. There are also numerous pointers to how male authority, when bolstered by the legal right to use corporal punishment to enforce moral codes of honour, may well have become institutionalised within interpersonal relationships, leading to the high rates of intimate partner violence that continue to exist today.

As we have emphasised from the outset, the coloniality of gender is neither a linear imposition of a Western gender system nor has it all been damaging. With the arrival of education, development, and health programmes, women are drawn into the public sphere, begin to take on positions of responsibility, earn their own money, and learn that they have the right to be free from violence, to leave the home without requesting permission, to study, and to take paid employment. This top-down process of coloniality has brought huge benefits to the lives of women and of men, in what is a continual process of gendered social change. Lugones’ idea that the colonial-modern system was ‘imposed’ upon them would suggest that Indigenous and colonised people played no part in the process, an idea that we strongly refute. This was a messy process of back and forth, albeit one from which gender, over the course of several centuries, took the form of patriarchy. However, as we have evidenced with both archive and ethnographic data, towards the end of the 20th century women’s health, education, and welfare gained in importance and both *teiwari* and Wixaritari women began to occupy prominent roles in the community. With this came the most recent negotiation, the improvement of women’s position in Wixárika communities, in interpersonal relationships, and in their access to services and education, improving the lives of women. This most recent ‘contact zone’ is one in which the coloniality of gender has improved the lives of women as global and modern ideas of gender equality have trickled down into the lives and social organisation in Wixárika communities.

## Notes

1 Director of Centro Coordinador Indigenista in Mezquitic, Jalisco, 1998–2005.

2 From the mid-19th century onwards the Mexican state promoted the idea of a ‘mestizo’ country, that meant a person from a mixed race who no longer identified with an Indigenous ancestor and who only spoke Spanish.
